# Efficacy and safety of artemether-lumefantrine for the treatment of uncomplicated malaria and prevalence of *Pfk13* and *Pfmdr1* polymorphisms after a decade of using artemisinin-based combination therapy in mainland Tanzania

**DOI:** 10.1186/s12936-019-2730-1

**Published:** 2019-03-21

**Authors:** Deus S. Ishengoma, Celine I. Mandara, Filbert Francis, Eldin Talundzic, Naomi W. Lucchi, Billy Ngasala, Abdunoor M. Kabanywanyi, Muhidin K. Mahende, Erasmus Kamugisha, Reginald A. Kavishe, Florida Muro, Ally Mohamed, Renata Mandike, Sigsbert Mkude, Frank Chacky, Lynn Paxton, George Greer, Chonge A. Kitojo, Ritha Njau, Troy Martin, Meera Venkatesan, Marian Warsame, Eric S. Halsey, Venkatachalam Udhayakumar

**Affiliations:** 1 National Institute for Medical Research, Tanga Research Centre, Tanga, Tanzania; 2 Malaria Branch, Division of Parasitic Diseases and Malaria, Centers for Disease Control and Prevention, Atlanta, GA USA; 3 Department of Parasitology, School of Public Health, Muhimbili University of Health and Allied Sciences, Dar es Salaam, Tanzania; 4 Department of Women’s and Children’s Health, International Maternal and Child Health (IMCH), Uppsala University, Uppsala, Sweden; 5 Ifakara Health Institute, Dar es Salaam, Tanzania; 6 Catholic University of Health and Allied Sciences/Bugando Medical Centre, Mwanza, Tanzania; 7 Kilimanjaro Christian Medical Centre/Kilimanjaro Christian Medical University College, Moshi, Tanzania; 8 National Malaria Control Programme, Ocean Road/Luthuli Avenue (NIMR Complex), Dar es Salaam, Tanzania; 9 U.S. President’s Malaria Initiative, U.S. Agency for International Development, U.S. Embassy, Dar es Salaam, Tanzania; 10 World Health Organization Country Office, Dar es Salaam, Tanzania; 11 HIV Vaccine Trials Network, Fred Hutch Cancer Research Center, Seattle, WA USA; 12 U.S. President’s Malaria Initiative, U.S. Agency for International Development, Washington, DC USA; 13 Global Malaria Programme, World Health Organization, 20 Avenue Appia, 1211 Geneva 27, Switzerland; 14 U.S. President’s Malaria Initiative, Centers for Disease Control and Prevention, Atlanta, GA USA; 15 Present Address: Gothenburg University, Gothenburg, Sweden

**Keywords:** Efficacy, Safety, Artemether-lumefantrine, Falciparum malaria, Tanzania

## Abstract

**Background:**

The World Health Organization recommends regular therapeutic efficacy studies (TES) to monitor the performance of first and second-line anti-malarials. In 2016, efficacy and safety of artemether-lumefantrine (AL) for the treatment of uncomplicated falciparum malaria were assessed through a TES conducted between April and October 2016 at four sentinel sites of Kibaha, Mkuzi, Mlimba, and Ujiji in Tanzania. The study also assessed molecular markers of artemisinin and lumefantrine (partner drug) resistance.

**Methods:**

Eligible patients were enrolled at the four sites, treated with standard doses of AL, and monitored for 28 days with clinical and laboratory assessments. The main outcomes were PCR corrected cure rates, day 3 positivity rates, safety of AL, and prevalence of single nucleotide polymorphisms in *Plasmodium falciparum* kelch 13 (*Pfk13*) (codon positions: 440–600) and *P. falciparum* multi-drug resistance 1 (*Pfmdr1*) genes (codons: N86**Y**, Y184**F** and D1246**Y**), markers of artemisinin and lumefantrine resistance, respectively.

**Results:**

Of 344 patients enrolled, three withdrew, six were lost to follow-up; and results were analysed for 335 (97.4%) patients. Two patients had treatment failure (one early treatment failure and one recrudescent infection) after PCR correction, yielding an adequate clinical and parasitological response of > 98%. Day 3 positivity rates ranged from 0 to 5.7%. Common adverse events included cough, abdominal pain, vomiting, and diarrhoea. Two patients had serious adverse events; one died after the first dose of AL and another required hospitalization after the second dose of AL (on day 0) but recovered completely. Of 344 samples collected at enrolment (day 0), 92.7% and 100% were successfully sequenced for *Pfk13* and *Pfmdr1* genes, respectively. Six (1.9%) had non-synonymous mutations in *Pfk13*, none of which had been previously associated with artemisinin resistance. For *Pfmdr1,* the N**F**D haplotype (codons N86, 184**F** and D1246) was detected in 134 (39.0%) samples; ranging from 33.0% in Mlimba to 45.5% at Mkuzi. The difference among the four sites was not significant (p = 0.578). All samples had a single copy of the *Pfmdr1* gene.

**Conclusion:**

The study indicated high efficacy of AL and the safety profile was consistent with previous reports. There were no known artemisinin-resistance *Pfk13* mutations, but there was a high prevalence of a *Pfmdr1* haplotype associated with reduced sensitivity to lumefantrine (but no reduced efficacy was observed in the subjects). Continued TES and monitoring of markers of resistance to artemisinin and partner drugs is critical for early detection of resistant parasites and to inform evidence-based malaria treatment policies.

*Trial Registration* ClinicalTrials.gov NCT03387631

## Background

Despite a decline of malaria burden over the past decade, malaria remains a major public health threat [1, 2]. An estimated 435,000 deaths and over 219 million cases were reported in 2017 (an increase of 5 and 3 million cases compared to 2015 and 2016, respectively) [1]. Approximately 93% of the deaths and 92% of the cases were from sub-Saharan Africa, with the majority occurring in children under 5 years of age or pregnant women [1]. Malaria control relies on a handful of interventions, including prompt and effective treatment with anti-malarials [3], a strategy threatened by parasite resistance to anti-malarial drugs in Southeast Asia [4].

Artemisinin-based combination therapy ACT is recommended by the World Health Organization (WHO) for the treatment of uncomplicated malaria caused by *Plasmodium falciparum* [5]. The currently recommended combinations include artemether-lumefantrine (AL), artesunate-amodiaquine (ASAQ), artesunate-mefloquine, dihydroartemisinin-piperaquine (DP) and artesunate–sulfadoxine/pyrimethamine (AS + SP) [6]. Tanzania introduced AL as its first line drug for the treatment of uncomplicated falciparum malaria in 2006 [7], and it remains the sole first-line treatment recommended in the country [8]. Studies conducted in the East African countries of Tanzania [9–12], Kenya [13], Uganda [14], Rwanda [15], Burundi [16], and in other African countries [17–19] have shown that AL, as well as other artemisinin-based combinations, such as ASAQ and DP (which are first or second-line therapies in other African countries) have high therapeutic efficacy and are well tolerated with minimal adverse effects. To date, there are no reports of clinically significant artemisinin resistance in Africa, and the laboratory correlates of resistance in Southeast Asia, delayed parasite clearance [20] or *Plasmodium falciparum kelch 13* (*Pfk13*) gene mutations [21–23] are rare in Africa. Studies from Southeast Asia have demonstrated resistance to partner drugs, including piperaquine [22, 24], yielding parasites resistant to both components of ACT, a scenario fortunately not currently observed in Africa.

Due to the threat of artemisinin drug resistance, WHO recommends regular surveillance (biennial) to monitor the performance of anti-malarials in malaria endemic countries. Furthermore, the WHO recommends molecular surveillance based on known polymorphisms (including copy number variations) in the *P. falciparum* genome as markers of resistance to artemisinins and partner drugs of the currently used artemisinin-based combinations. A number of single nucleotide polymorphisms (SNPs) in the *Pfk13* gene have been shown to confer resistance to artemisinins [25, 26]; and a number of mutations (codons F446**I**, N458**Y**, M476**I**, Y493**H**, R539**T**, I543**T**, P553**L**, R561**H** and C580**Y**) have been validated as markers of parasite resistance to artemisinins in Southeast Asia [27]. Polymorphisms in the *P. falciparum* multidrug resistance 1 (*Pfmdr1*) gene appear to be associated with decreased susceptibility to lumefantrine [28, 29]. Gene duplication leading to increased *Pfmdr1* copy numbers has been suggested to be associated with reduced susceptibility to lumefantrine [30].

In Tanzania, the National Malaria Control Programme (NMCP) and its partners have been implementing therapeutic efficacy studies (TESs) to monitor the efficacy and safety of different anti-malarials [31, 32], including currently used artemisinin-based combinations or of potential use in the country [9–12]. The studies have been conducted at eight sentinel sites located in regions with different transmission intensity, with some in border areas possessing potential for introduction of parasites from neighbouring countries [31]. Independent researchers have also conducted similar studies at the NMCP sentinel sites or other study areas [9]. These data supported changes in national anti-malarial treatment guidelines to replace chloroquine with SP in 2001 [33] and SP with AL in 2006 [7]. The study reported herein was carried out to assess the efficacy and safety of AL for the treatment of uncomplicated falciparum malaria, and the prevalence of molecular markers known to be associated with artemisinin resistance and reduced susceptibility to the partner drug (lumefantrine) after using AL in Tanzania for 10 years.

## Methods

### Study sites

**Fig. 1:**
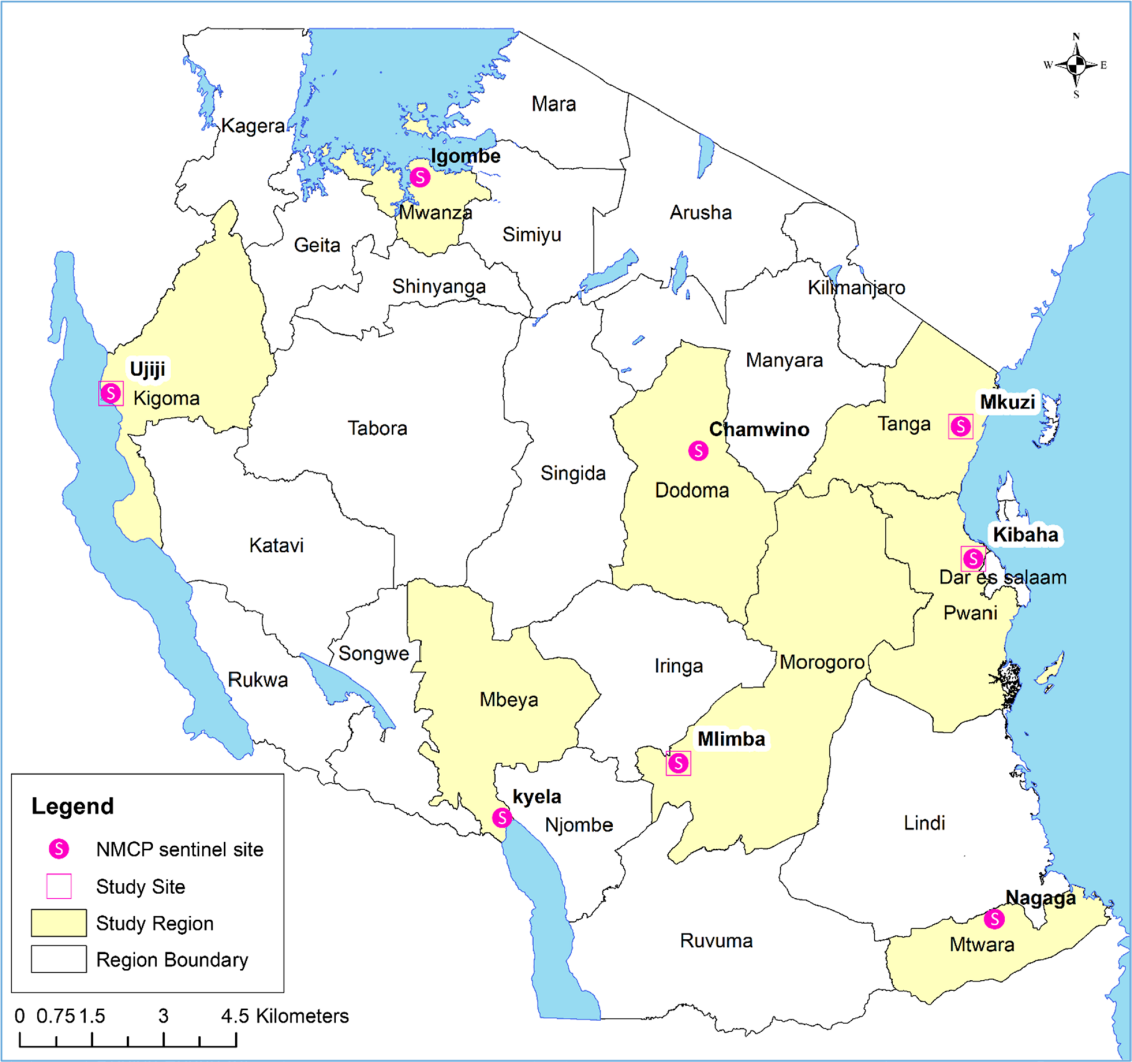
Regional map of Tanzania with eight National Malaria Control Programme sentinel sites, with the four sites covered in the 2016 study marked with white squares

This study was carried out at four of the eight NMCP sentinel sites (Kibaha—Coast region, Mkuzi—Tanga, Mlimba—Morogoro, and Ujiji—Kigoma) between April and September 2016. The study sites (Fig. 1) have been NMCP sentinel sites for monitoring of anti-malarial efficacy since 1997 [31, 32].

In Kibaha, the study was conducted at Yombo Health Centre, which is located in Kibaha district of Coastal region (Pwani), about 100 km west of Dar es Salaam (the commercial capital of Tanzania). Kibaha has low malaria transmission (< 10%) as reported in previous population surveys [34–36]. Malaria transmission in Kibaha occurs throughout the year and peaks during or just after the rainy season.

Mkuzi health centre is located in Muheza district of Tanga region, in northeastern Tanzania. The malaria epidemiological profile of Muheza district has been well characterized and a detailed description given elsewhere [37, 38]. Mkuzi is also one of the sentinel sites with low malaria transmission in Tanzania with parasite prevalence of < 5%, as reported in a 2017 national survey [39]. Details of the site as well as Muheza have been recently covered elsewhere [40].

Ujiji health centre is located in Kigoma urban district of Kigoma region, on the eastern shores of Lake Tanganyika in the northwestern part of Tanzania. Despite a decline in malaria transmission reported from 2007 onwards [34–36, 39], overall malaria transmission in Kigoma has increased in the past decade. According to recent national surveys, parasite prevalence increased from its 2007 level of 19.6% to 38.1% in 2016, followed by a decline to 24.4% in 2017, which was the highest prevalence in the country [34–36, 39]. Further details about Ujiji site have been given elsewhere [40].

Mlimba health centre is located in Kilombero district of Morogoro region and the areas around this site have been extensively studied under the Ifakara Health Institute demographic surveillance system over the past two decades [41–43]. Kilombero had high malaria transmission, with parasite prevalence of about 70.0% and entomological inoculation rates (EIR) of > 300 infectious bites per person per year in the 1990s [44]. However, malaria transmission in Kilombero has recently declined due to different interventions, and it is now a low transmission area (prevalence < 10% in 2017) [39].

### Study design and study population

This was a single-arm prospective in vivo study designed to assess the therapeutic efficacy and safety of AL for the treatment of uncomplicated falciparum malaria and the markers of artemisinin and lumefantrine resistance. Malaria transmission has been decreasing in some of the sites compared to the past and, therefore, thresholds of parasitaemia for low transmission areas recommended by WHO (between 250 and 200,000 asexual parasites/µl) were used. The age of study participants (6 months to 10 years) was also broadened [45, 46].

### Sample size estimation

Per the WHO protocol, sample size estimates assumed 5% of the enrolled patients would have treatment failure after treatment with AL. At a confidence level of 95% and an estimate precision of 5%, a minimum sample size of 73 patients was required to detect a failure rate ≤ 5% [46]. With a 20% increase to allow for loss to follow-up and withdrawals during the 28-days of follow-up, 88 patients were targeted per site, giving a total of 352 at the four sites.

### Screening and recruitment

At each of the study sites, potential participants were screened at the outpatient departments using malaria rapid diagnostic tests and microscopy as previously described [40]. Patients were eligible for enrolment if they were aged 6 months to 10 years, had fever at presentation (axillary temperature ≥ 37.5 °C) and/or history of fever in the last 24 h, a positive rapid diagnostic test, and parasitaemia of 250 to 200,000 asexual parasites/µl by microscopy. Other inclusion and exclusion criteria were assessed according to the WHO protocol [46]. Patients who could not be enrolled in the study received appropriate treatment according to national guidelines [8]. In addition to the malaria parasite identification, dried blood spots (DBS) on filter paper (Whatmann No. 3, GE Healthcare Life Sciences, PA, USA) were collected for parasite genotyping (to distinguish recrudescence from new infections) and for analysis of markers of artemisinin and lumefantrine resistance. Microscopy was performed during each subsequent visit to determine infection status, species, and parasite density.

### Examination of malaria parasites by microscopy

Two blood slides were collected, and one of the slides was stained with 10% Giemsa for 10–15 min and examined by microscopy to detect presence of and an estimated density of malaria parasites. The second blood slide was stained with 3% Giemsa for 30–45 min and used to determine the actual parasite density, species, and presence of gametocytes. Parasitaemia was measured by counting the number of asexual parasites against 200 leucocytes in thick blood films and detection of the different parasite species was done on thin films. Parasite density per µl of blood was calculated by multiplying the total count by 40, assuming that 1 µl of blood had a mean count of 8000 leucocytes [26]. When more than 500 parasites were identified before counting 200 leucocytes, counting was stopped and parasitaemia was calculated using the actual number of leucocytes counted. A blood slide was declared negative when examination of 100 high power fields did not reveal the presence of malaria parasites. For quality control, each slide was re-examined by a second microscopist, and those with discrepant results were re-examined by a third microscopist. Any further disagreement was resolved by a team of three microscopists, who examined the same slide at the same time. Final parasitaemia was calculated as the average between the two closest readings.

### Treatment and clinical monitoring during follow-up

Enrolled patients were treated with AL (Coartem^®^, Beijing Novartis Pharma Ltd, Beijing China; provided by WHO) for 3 days. Weight-based dosing based on one of three ranges (5–14 kg, 15–24 kg, or 25–35 kg) was done using a fixed dose combination of 20 mg of artemether and 120 mg lumefantrine per tablet, thus patients were given one, two or three tablets from smallest to heaviest, respectively. A full course of AL consisted of 6 doses given twice daily (8 h apart on day 0 and 12-h on days 1 and 2). Patients were observed for 30 min to ensure they did not vomit the study drugs. If vomiting occurred, a repeat dose was given after vomiting stopped. Any patient who persistently vomited the study medication was withdrawn and treated with quinine (injection/intravenous) or artesunate injection according to the national guidelines for management of complicated and severe malaria [8]. Paracetamol was given to all patients with body temperature ≥ 38 °C. All (morning and evening) doses were administered orally at the health facility under direct observation of a study nurse but without nutritional supplements. Patients living far from the study health facilities were retained in the wards for the evening and morning doses of the drugs, while those staying close were provided with transport to the facilities for the evening doses.

Follow-up was done for 28 days with scheduled visits on days 1, 2, 3, 7, 14, 21, and 28 or at any other time (unscheduled visit) when patients felt unwell. Parents/guardians were informed and encouraged to bring their children back to the clinic whenever they were unwell without waiting for scheduled visits. Patients who did not show up for their scheduled visits by mid-day were visited at home by a member of the study team and asked to come to the health facility. If a patient had travelled and could not be traced for scheduled follow-up, he/she was classified as lost to follow-up. During the visits, both clinical and parasitological assessments were performed and DBS were also collected. Patients with recurrent infections occurring on day 7 and afterwards were treated with quinine (tablets, injection/intravenous) or artesunate injection based on clinical presentation as per WHO protocol [46].

### Safety assessment

The safety of AL was monitored by both passive and active methods through interviews with parents/guardian and clinical/laboratory assessments during the 28 days of follow-up. This enabled investigators to capture and record adverse events (AEs) or severe adverse events (SAEs) that occurred after treatment. During scheduled visits, parents/guardians were directly interviewed and asked to report the occurrence, nature, and incidence of any events occurring at home between the follow-up visits. Clinicians took a history, observed patients, and performed a clinical examination during follow-up visits at the study sites. Laboratory tests were appropriately requested and done to determine and capture AEs/SAEs. The reported/captured events were recorded in respective case report forms for each follow-up visit. Any SAE occurring during the study was reported by the principal investigator to the sponsor (National Institute for Medical Research—NIMR), NMCP, and the Tanzanian Medical Research Coordinating Committee (MRCC) of NIMR (which is the Tanzanian national ethics committee) within 24 h of its occurrence. Reporting of SAEs was done regardless of whether the principal investigator considered the events to be related to the investigated drug or not. Patients with AEs or SAEs were thoroughly assessed and managed accordingly, and the events were also assessed to determine their association with the study drugs. According to the WHO protocol [46], an AE was defined as any unfavourable, unintended sign, symptom, syndrome or disease that develops or worsens with the use of a medicinal product, regardless of whether it is related to the medicinal product. An SAE was also defined as any untoward medical occurrence that at any dose may lead to either death, life threatening condition, hospitalization or prolongation of hospitalization, significant disability, or incapacity.

### Sample processing and molecular analysis

Molecular analysis was performed on all samples collected upon enrolment (day 0) and during follow up in the case of treatment failure. Parasite genomic DNA was extracted from DBS using QIAamp blood mini-kits (Qiagen GmbH, Hilden, Germany) according to the manufacturer’s instructions. Molecular markers of anti-malarial drug resistance and microsatellite markers were analysed at the Centers for Disease Control and Prevention (CDC) Malaria Laboratory in Atlanta, USA. Sanger sequences generated in this study were analysed using the Geneious software package (Biomatters, Inc., San Francisco, CA). Raw sequence reads were cleaned using default settings, and reads with high-quality scores (the percentage of high-quality bases) below 70% were discarded from further analysis. The *Pfk13* propeller domain (codon positions: 440–600) and *Pfmdr1* (codon positions: 86, 184 and 1246) were analysed for SNPs. SNPs were called only if they fit the following criteria: (i) they were found on both the forward and reverse reads, (ii) they had a *p* - value of < 0.0001 (*p*-value represents the probability of a sequencing error resulting in observing bases with at least the given sum of qualities), and (iii) they had a minimum strand bias *p*-value of < 0.0005 when exceeding 65% strand bias, as some errors from sequencing machines are more likely to happen on nearby upstream bases. Mixed-infection and/or heterozygous calls were excluded from the analysis. The 3D7 *Pfk13* and *Pfmdr1* were used as reference sequences. Detection of *Pfmdr1* copy number variants was performed using an Agilent Mx3005 real-time PCR machine (Agilent Technologies, California, USA) according to previously described protocols [47, 48].

Samples from 65 patients with recurrent infections were analysed to determine genetic diversity in the study populations using six neutral microsatellite markers (TA1 on Chromosome 6, Poly-α on chromosome 4, PfPK2 on chromosome 12, 2490 on chromosome 10, C2M34-313 on chromosome 2 and C2M69-383 on chromosome 3) by nested PCR for all except C2M34-313 and C2M69-383 (which were analysed with a single step PCR). Fragment size was measured by capillary electrophoresis on ABI 3033 (Applied Biosystems) and scored using GeneMarker^®^ V2.6.3 (SoftGenetic, LLC, PA, USA) [48]). Paired samples (day 0 and parasites collected on or after day 7) were analysed to distinguish recrudescent from new infections by comparing alleles of samples collected at enrolment (on day 0) with those collected on the day of recurrent infection as previously described [49, 50]. A recrudescent infection was confirmed if there was a perfect match of alleles at all successfully genotyped microsatellite markers while any mismatch was reported as a new infection. Cases with genotyping failure in either day 0 or recurrent samples (or both) at all markers were reported as non-determined (unknown PCR) and excluded from analysis of PCR corrected treatment outcome.

### Outcome classification

The primary end point was PCR corrected parasitological cure on day 28 as per WHO protocol [46], while secondary end points included parasitaemia on day 3 post-treatment, occurrence of AEs/SAEs, and molecular markers of drug resistance in *Pfmdr1* and *Pfk13* genes. Treatment outcomes were classified as either early treatment failure (ETF), late clinical failure (LCF), late parasitological failure (LPF), or adequate clinical and parasitological response (ACPR) before and after PCR correction; based on per protocol method and Kaplan–Meier analysis.

### Ethical considerations

Ethical clearance was obtained from MRCC of NIMR, while permission to conduct the study at the health facilities was sought in writing from the relevant regional and district medical authorities. Ethical clearance form CDC was not required because the assessments done at the CDC Malaria Laboratory, using samples without linked identifiers, were determined by the CDC Center of Global Health’s Human Research Protection Coordinator to not constitute engagement in human subjects’ research. Detailed information of the study and benefits as well as its risks were explained to each study participant. Oral and written informed consent were obtained from parents or guardians of all patients before they were screened for possible inclusion into the study. The study was retrospectively registered at ClinicalTrials.gov, No. NCT03387631 (on 2nd January 2018) because of problems within the system which did not allow to register it before the study was launched.

### Data management and analysis

Data were entered into a Microsoft Access database at the study sites followed by a second entry which was done centrally at NIMR Tanga Centre after the end of data collection. The data were later validated, cleaned, and analysed using STATA for Windows version 11 (STATA Corporation; TX, USA). Descriptive statistics such as percentages, mean, median, standard deviation, and range were reported as appropriate. In order to automatically generate the treatment outcomes (based on per protocol and Kaplan–Meier analysis), the data were appropriately formatted and transferred to the WHO Excel software template [51]. Baseline characteristics, primary outcomes, and secondary outcomes were compared among the four sites. Continuous variables, such as log_10_ transformed parasite density at enrolment and age, among the four sites were compared using *t* test or analysis of variance—ANOVA (for normally distributed data) or Mann–Whitney U/Kruskal–Wallis test (non-parametric tests for non-normally distributed data). The prevalence of different haplotypes or SNPs in the *Pfmrd1* and *Pfk13* genes as well as *Pfmdr1* copy numbers were reported. For the *Pfmrd1* gene, the analysis focused on the three SNPs (N86**Y**, Y184**F** and D1246**Y**) and their corresponding haplotypes (particularly N**F**D), which have been associated with reduced susceptibility to lumefantrine [28, 29]. Logistic regression was used to assess the odds of selection from lumefantrine sensitive (**Y**Y**Y**) to resistant haplotype (N**F**D) on day zero and day of recurrence with adjustment for age of patients and the study site (to account for potential differences in parasite populations attributable to variations in geographic locations). For all statistical tests and comparisons, a p-value < 0.05 (two tailed) was considered to be significant.

## Results

### Baseline characteristics of enrolled children

**Fig. 2:**
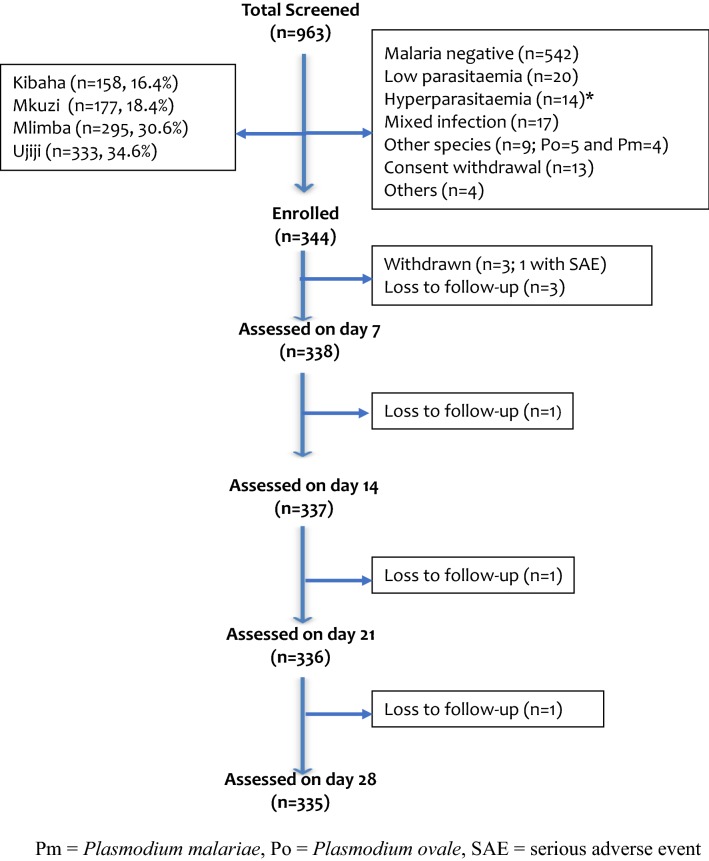
Trial profile of the 2016 therapeutic efficacy study showing the flow of patients during screening, enrolment and follow-up. *1 patient from Ujiji had hyperparasitaemia and mixed infection (counted only once in this category)

A total of 963 children were screened between April and October 2016. All sites recruited 88 children except Kibaha, which enrolled 80, making a total of 344 enrolled at the four sites (Fig. 2). Table 1 shows the baseline characteristics of enrolled patients. More boys (57.0%) were enrolled than girls, but the difference among the sites was not significant (p = 0.962). Overall, Mlimba recruited significantly younger children compared to other sites, while children with the highest age were recruited at Kibaha (p < 0.001). The proportion of children under 5 years of age recruited at the two sites of Kibaha and Mkuzi was significantly lower compared to Mlimba and Ujiji (p < 0.001). The average axillary temperature recorded at enrolment (on day 0) was similar at all sites. The geometric mean parasite density (asexual parasites/µl) was significantly higher at Ujiji (p < 0.001) compared to the other sites; patients enrolled at Kibaha had the lowest parasitaemia (Table 1).

**Table 1:** Numbers of patients screened and baseline characteristics of patients enrolled in the therapeutic efficacy study at four sentinel sites in 2016

Variable	Kibaha	Mkuzi	Mlimba	Ujiji	Overall
Screened	158	177	295	333	963
Enrolled (%)	80 (50.6)	88 (49.7)	88 (30.3)	88 (26.4)	344 (35.7)
Age in years, mean (SD)^a^	6.5 (2.7)	5.7 (2.8)	3.4 (2.3)	4.6 (2.9)	5.0 (2.9)
Children < five years of age, n (%)†	29 (36.3)	36 (40.9)	66 (75.0)	58 (65.9)	189 (54.9)
Sex (male), n (%)	46 (57.5%)	49 (55.7%)	49 (55.7%)	52 (59.1%)	196 (57.0)
Weight (kg), mean (SD)^b^	19.1 (5.6)	17.4 (5.0)	13.1 (4.3)	14.9 (5.1)	16.1 (5.5)
Height in cm, median (IQR)^c^	114.0 (98.5, 128.3)	111.0 (92.1, 121.0)	85.5 (77.0, 99.8)	97.0 (86.0, 111.8)	100.0 (85.0, 117.8)
Temperature in °C, mean (SD)	37.8 (1.4)	37.7 (1.1)	37.6 (1.3)	37.7 (1.4)	37.7 (1.3)
Parasitaemia* (95% CI)^a^	9403 (5901–14,984)	32,357 (23,393–44,756)	27,720 (20,706–37,108)	41,106 (30,356–55,664)	24,806 (20,701–29,726)

°C: degree Celsius; parasitaemia*: geometric mean parasite density (asexual parasites/µl); IQR: interquartile range; n: number of patients; SD: standard deviation; 95% CI : 95% confidence interval. † p < 0.001; ^a^the mean was significantly different between all paired comparison of the sites (p ≤ <0.031) except for Kibaha *vs* Mkuzi (p = 0.300); ^b^the mean weight was significantly different between all paired comparison of the sites (p ≤ <0.005) except for Kibaha *vs* Mkuzi (p = 0.200), and Mlimba vs Ujiji (p = 0.120); ^c^the mean height was significantly different between all paired comparison of the sites (p < 0.001) except for Kibaha *vs* Mkuzi (p = 0.138), and Mkuzi vs Ujiji (p = 0.068)

### Efficacy outcomes

Among the 344 patients enrolled, six (1.7%) were lost to follow-up and three (0.9%) withdrew, leaving 335 (97.4%) patients that reached the study end points who were used in per protocol analysis (Fig. 1 and Table 2). In the Kaplan–Meier analysis, lost and withdrawn cases were included in the analysis until the last day seen. Based on the PCR uncorrected data, one patient (from Ujiji site) had ETF (0.3%), 21 (6.3%) had LCF, 47 (14.0%) had LPF, and 266 (79.4%) had ACPR (Table 2). After PCR correction, only one patient (0.4%) had LCF, with a recrudescent infection reported on day 28; together with one patient with ETF (0.4%), the overall PCR corrected ACPR was 99.3% (≥ 98.4% at each site). Eleven (3.2%) patients had parasitaemia on day 3 post-treatment, and no patients from Kibaha had parasites on day 3 while the highest positivity rates (5.7%) was reported at Mkuzi (Table 2).

**Table 2:** Measures of therapeutic efficacy of AL before and after PCR correction

Outcome	Kibaha n (%; 95% CI)	Mlimba n (%; 95% CI)	Mkuzi n (%; 95% CI)	Ujiji n (%; 95% CI)	Total n (%; 95% CI)
PCR uncorrected					
Parasitaemia on day 3	0 (0; 0–4.5)	2 (2.3; 0.3–8.1)	5 (5.7; 1.9–12.9)	4 (4.7; 1.3–11.5)	11 (3.3;1.7–5.8)
ETF	0 (0; 0–4.7)	0 (0; 0–4.2)	0 (0; 0–4.2)	1 (1.1%; 0–6.2)	1 (0.3; 0–1.7)
LCF	12 (15.8; 8.4–26.0)	2 (2.3; 0.3–8.1)	3 (3.5; 0.7–9.9)	4 (4.6; 1.3–11.4)	21 (6.3; 3.9–9.4)
LPF	6 (7.9; 3.0–16.4)	11 (12.8; 6.6–21.7)	11 (12.8; 6.6–21.7)	19 (21.8; 13.7–32.0)	47 (14.0; 10.5–18.2)
ACPR	58 (76.3; 65.2–85.3)	73 (84.9; 75.5–91.7)	72 (83.7; 74.2–90.8)	63 (72.4; 61.8–81.5)	266 (79.4; 74.7–83.9)
Total for per protocol	76	86	86	87	335
Withdrawn	0 (0.0%)	1 (1.1)	1 (1.1)	1 (1.1)	3 (0.9%)
Lost to follow-up	4 (5.0%)	1 (1.1)	1 (1.1)	0 (0)	6 (1.7%)
Total at baseline	80	88	88	88	344
PCR corrected					
ETF	0 (0; 0–6.2)	0 (0; 0–4.9)	0 (0; 0–4.9)	1 (1.6;)	1 (0.4; 0–2.1)
LCF	0 (0; 0–6.2)	0 (0; 0–4.9)	1 (1.4:)	0 (0; 0–5.6)	1 (0.4; 0–2.1)
LPF	0 (0; 0–6.2)	0 (0; 0–4.9)	0 (0; 0–4.9)	0 (0; 0–5.6)	0 (0; 0–1.4)
ACPR	58 (100; 93.8–100)	73 (100; 95.1–100)	72 (98.6; 92.6–100)	63 (98.4; 91.6–100)	266 (99.3; 97.3–99.9)
Total for per protocol	58	73	73	64	268
Withdrawn/lost to follow-up	4 (5.0%)	2 (2.2)	2 (2.2)	1 (1.1)	9 (2.6)
Re-infection	15 (18.8)	11 (12.5)	9 (10.2)	20 (22.7)	55 (16.0)
Unknown PCR^a^	3 (3.4)	2 (0.6)	4 (1.2)	3 (3.4)	12 (3.5)
Total at baseline	80	88	88	88	344
KM cumulative success rate	58 (100)	73 (100)	72 (98.6)	63 (98.4)	266 (99.3)

ACPR: adequate clinical and parasitological response; ETF: early treatment failure; LCF: late clinical failure; LPF: late parasitological failure; PP: number of patients involve in the per protocol analysis; KM: Kaplan–Meier

^a^The samples could not be resolved after PCR because of inconsistent PCR results

### Safety outcomes

The commonly reported adverse events included cough, abdominal pain, vomiting, and diarrhoea. Most events were reported at Mkuzi site (Table 3). The time periods when these events occurred during follow-up are presented in Supplemental Table 1. Two patients had SAEs on day 0, including one patient (9 years, Female) from Mkuzi who died after the first dose of AL and a second patient (3.5 years, Male) from Ujiji who was hospitalized after the second dose of AL. Further assessment showed that the child from Ujiji was brought back with severe malaria, high fever (temperature = 39.5 °C), vomiting, and convulsions after the second dose of AL. This child was treated with injectable artesunate (intravenous) and later transitioned to AL tablets and recovered completely. However, the child from Mkuzi succumbed to severe malaria with high fever (axillary temperature = 39.2 °C) and convulsions after the first dose of AL and died immediately after arrival in the ward before any further treatment could be given. The child had no danger signs at enrolment and the initial parasitaemia was 16,160 asexual/µl. The axillary temperature at screening was 37.1 °C and the child walked by herself to the facility at the initial consultation. Post-mortem examination could not be performed because the deceased was buried the following day based on the family’s decision. The actual cause of death was not established and could not be ascertained if it was associated with the medication.

**Table 3:** Number and proportion of patients with at least one adverse event

Adverse events	Kibaha (n = 80)	Ujiji (n = 88)	Mkuzi (n = 88)	Mlimba (n = 88)	Total (n = 344)
Cough	0 (0%)	12 (13.6%)	31 (35.2%)	1 (1.1%)	44 (12.8%)
Abdominal pain	2 (2.5%)	1 (1.1%)	12 (13.6%)	0 (0%)	15 (4.4%)
Vomiting	3 (3.8%)	0 (0%)	9 (10.2%)	1 (1.1%)	13 (3.8%)
Diarrhoea	0 (0%)	0 (0%)	3 (3.4%)	1 (1.1%)	4 (1.2%)
Headache	1 (1.3%)	0 (0%)	1 (1.1%)	0 (0%)	2 (0.6%)
Others	0 (0%)	10 (11.4%)	5 (3.4%)	3 (3.4%)	18 (5.2%)
Total	6 (7.5%)	23 (26.1%)	61 (69.3%)	6 (6.8%)	96 (27.9%)

### Molecular markers of anti-malarial drug resistance

**Fig. 3:**
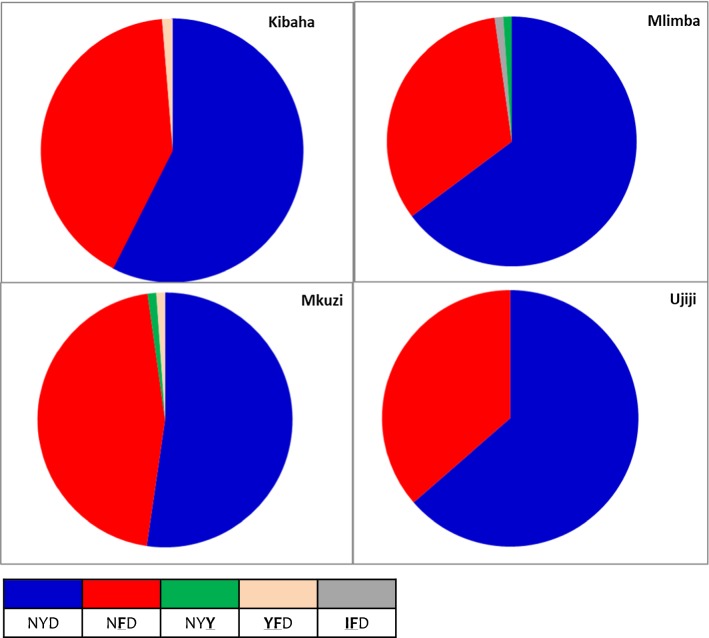
Prevalence of different haplotypes in the *Pfmdr1* gene in baseline samples collected at enrolment of study patients

Out of 344 samples collected at enrolment (on day 0), 92.7% (n = 319) and 100% (n = 344) were successfully sequenced for *Pfk13* and *Pfmdr1*, respectively. Seventeen samples (5.3%) had mutations in the *Pfk13* gene and only 6 (1.9%) samples had non-synonymous mutations, of which none were similar to previously reported SNPs associated with artemisinin resistance. The six *Pfk13* non-synonymous mutations included one patient from Mkuzi (R471**S**), two at Mlimba (A578**S** and E433**D**), and three at Ujiji (one with I416**V** and two with Q613**E**). All patients with parasitaemia on day 3 post-treatment had infections with *Pfk13* wild-type at enrolment. For *Pfmdr1*, codons N86**Y**, Y184**F** and D1246**Y** were analysed and the prevalence of the different SNPs are presented in Table 4. The N86 and D1246 polymorphisms were found at a prevalence of greater than 98.9% across the four sites. In comparison, the 184**F** mutant was present between 34.1% (Mlimba) and 46.6% (Mkuzi) in all four sites (Table 4), the differences were not significant (p > 0.310). The N**F**D *Pfmdr1* haplotype, which is possibly associated with decreased susceptibly to lumefantrine, was detected in 134 (39.0%) samples, and its prevalence ranged from 33.0% in Mlimba to 45.5% at Mkuzi, but there were no significant differences (p = 0.578) among the four sites (Fig. 3). Among 65 patients with recurrent infections (including 64 with new infections and one with a recrudescent infection), 39 (60.0%) had NYD (wildtype) haplotype at enrolment and 15 (38.5%) of these had selection to N**F**D haplotype (with mutations at codon 184**F**) during recurrent infections. Although patients with the N**F**D haplotype at baseline had more recurrent infections, the difference was not significant, even after adjusting for age and study site (adjusted OR = 1.17, 95% CI = 0.66–2.05, p = 0.594). Both patients with PCR-corrected treat failure (one with ETF and the second with a recrudescent infection) had N**F**D haplotype at enrolment. The overall median day of recurrent infection was 21 days (interquartile range, 21–28) and this was similar among the four sites. Recurrent infections among individuals with N**F**D haplotypes at enrolment had a median of 23 days compared to 21 days for NYD, but the difference was not statistically significant (p = 0.417). Analysis of *Pfmdr1* copy number variants was done on all 65 individuals with recurrent infections; all samples had a single copy of the gene.

**Table 4:** Day 0 prevalence of different *Pfmdr1* single nucleotide polymorphisms at the four sites covered in the therapeutic efficacy study in 2016

SNP	Kibaha	Mkuzi	Mlimba	Ujiji	Total
N86^a^	79 (98.8)	87 (98.9)	87 (98.9)	88 (100.0)	341 (99.1)
**184F**	34 (42.5)	41 (46.6)	30 (34.1)	32 (36.4)	137 (39.8)
D1246	80 (100.0)	87 (98.9)	87 (98.9)	88 (100.0)	342 (99.4)

^a^One sample from Mlimba had an N86**I** mutation while the others with mutations at this SNP possessed N86**Y** (2 samples, one each from Kibaha and Mkuzi)

## Discussion

This study reports high efficacy of AL (PCR corrected ACPR > 98%), suggesting that the ACT has retained its ability to treat uncomplicated malaria despite its use in Mainland Tanzania for more than 10 years. Previous studies conducted at these and other sites in Tanzania reported similarly high efficacies of AL [9–12]. However, given the high level of re-infections observed in the study areas, multiple interventions are warranted, including both curative and preventive approaches such as insecticide treated nets to reduce the burden of malaria. Studies conducted in different parts of Tanzania showed that about 30% of patients developed new infections during follow-up, and this was mainly attributed to high transmission of malaria [9]. Despite a decline in malaria transmission, which was reported from 2007 onwards [34–36, 39], this study still reported high rates of recurrent infections ranging from 15.1 to 27.6%, with the highest rates at Ujiji. This could be attributed to a resurgence of malaria, which indeed was reported in 2016, when parasite prevalence increased from its 2012 level of 9.5% to 14.8% nationally, with similar increases in the study regions [34, 36].

This study also showed that AL had a safety profile comparable to previous studies and was well tolerated with minimal AEs. Most of the AEs were minor and mainly reported at the two sites of Mkuzi and Ujiji. Studies conducted in Tanzania [9, 12] and elsewhere in Africa [18, 19, 52] reported similar safety profiles of AL when used for the treatment of uncomplicated falciparum malaria. A high number of cases reporting cough at Mkuzi could be attributed to weather conditions, which were relatively cold and rainy at the time of the study.

Two patients had serious adverse events on the day of presentation (day 0), with one child from Mkuzi dying after the first dose of AL. The cause of death was not established and its possible association with AL treatment could not be ascertained. A similar incident of death was reported in a TES which was conducted at Nagaga in 2012 [12], and such deaths reported in TES are unlikely to be caused by AL treatment. However, close monitoring of patients with a possibility of progressing to severe disease due to danger signs or high parasitaemia (> 100,000 asexual parasites/µl) has been recommended and will be undertaken in future studies.

Analysis of *Pfk13,* a molecular marker of artemisinin resistance [25], revealed that 17 (5.3%) samples had mutations at different locations of the gene, although the majority were synonymous changes and none of the non-synonymous mutations were similar to those reported to be associated with artemisinin resistance in Southeast Asia. The observed prevalence of *Pfk13* non-synonymous mutations in this study was higher than in a previous study which reported a prevalence of 1.2% [53]. All patients with parasites on day 3 had infections with parasites possessing wild type *Pfk13* at enrolment. These findings do not suggest that artemisinin resistance has emerged in Tanzania [21–23] and other malaria endemic areas in Africa, as previously reported [20]. However, they suggest that *Pfk13* mutations might be increasingly accumulating in Tanzanian parasite populations and thus continued surveillance will be required to monitor the trends of *Pfk13* polymorphisms and their possible association with reduced efficacy of artemisinins.

Analysis of the *Pfmdr1* gene showed that 39.0% of the parasites tested had the N86/Y184**F**/D1246 (N**F**D) haplotype, a combination associated with reduced susceptibility to lumefantrine in some studies [54]. This combination of *Pfmdr1* SNPs had higher prevalence than what was reported by previous studies in Mainland Tanzania [55] and Zanzibar [56]; however, this was not associated with increased risk of treatment failure following AL treatment. The significance of these findings is unclear but the SNPs may reflect the long-term use of AL in Tanzania and should be monitored in future studies.

Although studies have associated elevated *Pfmdr1* copy number with reduced susceptibility to lumefantrine [30, 47, 57], recent pooled analysis, however, failed to show such association (28) but supported an association with reduced sensitivity to mefloquine. The parasites analysed in this study had only one copy of *Pfmdr1* gene. This could be related to the fact that mefloquine has never been used as a routine first-line therapy in the study areas.

## Conclusion

The findings of this study showed that the efficacy of AL remains high in Tanzania despite the use of this combination for more than 10 years. The safety of AL was consistent with previous reports and no known artemisinin-resistance *Pfk13* mutations or amplification of the *Pfmdr1* gene were identified. A high prevalence of a *Pfmdr1* haplotype was detected but corresponding subjects with treatment failure were not found. Continued TESs and monitoring of markers of resistance to artemisinin and partner drugs is critical and should be sustained to facilitate early detection of resistant parasites and to inform evidence-based malaria treatment policies.

## Abbreviations

ACPR: adequate clinical and parasitological response
ACT: artemisinin-based combination therapy
AL: artemether-lumefantrine
ASAQ: artesunate + amodiaquine
AS + SP: artesunate + sulfadoxine/pyrimethamine
CDC: Centers for Disease Control and Prevention
DBS: dried blood spot
DNA: deoxyribonucleic acid
DP: Dihydroartemisinin–Piperaquine
ETF: early treatment failure
LCF: late clinical failure
LPF: late parasitological failure
MRCC: Medical Research Coordinating Committee
PARMA: US PMI-supported Antimalarial Resistance Monitoring in Africa Network
*Pfk13*: *Plasmodium falciparum* Kelch 13 gene
*Pfmdr1*: *Plasmodium falciparum* multi-drug resistance 1 gene
PMI: US President’s Malaria Initiative
NIMR: National Institute for Medical Research
NMCP: National Malaria Control Programme
PCR: polymerase chain reaction
SNP: single nucleotide polymorphism
TES: therapeutic efficacy study
USA: United States of America
WHO: World Health Organization
